# Lysosomal Activation Mediated by Endocytosis in J774 Cell Culture Treated with N-Trimethyl Chitosan Nanoparticles

**DOI:** 10.3390/molecules29153621

**Published:** 2024-07-31

**Authors:** Brenda I. Magaña-Trejo, Aldo Y. Tenorio-Barajas, Bulmaro Cisneros, Victor Altuzar, Sergio Tomas-Velázquez, Claudia Mendoza-Barrera, Efrain Garrido

**Affiliations:** 1 Departamento de Genética y Biología Molecular, Centro de Investigación y Estudios Avanzados del IPN, Mexico City 07360, Mexico; brenda.magana@cinvestav.mx (B.I.M.-T.);; 2 Facultad de Ciencias Físico Matemáticas, Benemérita Universidad Autónoma de Puebla, Puebla 72570, Mexico; 3 Departamento de Física, Centro de Investigación y Estudios Avanzados del IPN, Mexico City 07360, Mexico

**Keywords:** N-trimethyl chitosan, nanoparticles, encapsulation, endocytosis, immune system

## Abstract

Safety and effectiveness are the cornerstone objectives of nanomedicine in developing nanotherapies. It is crucial to understand the biological interactions between nanoparticles and immune cells. This study focuses on the manufacture by the microfluidic technique of N-trimethyl chitosan/protein nanocarriers and their interaction with J774 cells to elucidate the cellular processes involved in absorption and their impact on the immune system, mainly through endocytosis, activation of lysosomes and intracellular degradation. TEM of the manufactured nanoparticles showed spherical morphology with an average diameter ranging from 36 ± 16 nm to 179 ± 92 nm, depending on the concentration of the cargo protein (0, 12, 55 μg/mL). FTIR showed the crosslinking between N-trimethyl chitosan and the sodium tripolyphosphate and the α-helix binding loss of BSA. TGA revealed an increase in the thermal stability of N-trimethyl chitosan/protein nanoparticles compared with the powder. The encapsulation of the cargo protein used was demonstrated using XPS. Their potential to improve cell permeability and use as nanocarriers in future vaccine formulations was demonstrated. The toxicity of the nanoparticles in HaCaT and J774 cells was studied, as well as the importance of evaluating the differentiation status of J774 cells. Thus, possible endocytosis pathways and their impact on the immune response were discussed. This allowed us to conclude that N-trimethyl chitosan nanoparticles show potential as carriers for the immune system. Still, more studies are required to understand their effectiveness and possible use in therapies.

## 1. Introduction

Drug delivery systems, including nanoparticles (NPs), offer precise drug release and targeting within the body. Their significance lies in enhancing the formulation of existing drugs, which is not always optimal. This advancement is pivotal in diversifying pharmaceutical sales, as suggested by several studies [1,2]. Despite the development of additional formulations, many adjuvants are unsuitable for routine vaccines because of their toxicity and local side effects, such as deposit formation at the injection site. However, these adjuvants are crucial in enhancing immune responses, particularly in cases where antigenic variations are addressed, such as dengue (DENV) with multiple viral serotypes [3]. In addition, they play a critical role in expanding antibody responses and improving the efficacy of weak antigens. The choice of adjuvant can be tailored to desired effects, and combined adjuvants, such as liposomes or nanoparticles, enhance antigen presentation, with key characteristics such as size, charge, and shape impacting their effectiveness [4]. In particular, nanoparticle size can be controlled by synthesis techniques, such as microfluidics. Microdroplet generation in microfluidics assisted by emulsification can produce high-throughput monodisperse droplets by controlling the pressure of the input liquids, exploding the laminar flow created inside the selected channels and the nucleation-diffusion carried out outside the microfluidic cell [5,6]. The charge of adjuvants can be controlled by choosing the precursor material. Polymeric nanoparticles are particularly interesting because of their water-retention properties, charge, and biocompatibility [7]. One of the safest reported materials is chitosan; however, reports suggest its use is often complicated because of its low water solubility and high stability at acidic pH. Therefore, alternatives such as N-trimethyl chitosan (TMC) for nanoparticle synthesis confer higher stability and water solubility and enhance their electrostatic capabilities [8,9].

Polymeric nanoparticles have been reported in J774 cells to assess lysosomal activation. Additionally, TMC nanoparticles have been used as adjuvants and carriers for immune system activation in vivo. This involved assessing antibody production related to proteins loaded onto the nanoparticles, ranging from simple proteins, such as ovalbumin, to complete DENV viral particles. One crucial aspect to highlight is enhancing TMC carrier capacity and the potential use of nanoparticles in less conventional administration routes, such as the intranasal route. This is attributed to the physicochemical properties of TMC, which enhance the metabolism of the carried proteins [8,10,11].

Thus, cell-particle interactions are fundamental for developing carriers of therapeutic interest. In this study, nanoparticles based on N-trimethyl chitosan were fabricated by using microfluidics [6], and their morphology, average diameter, composition, and thermal and surface properties were characterized using transmission electron microscopy, Fourier transform infrared spectroscopy, thermogravimetric, and X-ray electron spectroscopy analyses. The effects of N-Trimethyl chitosan nanoparticles on HaCaT and J774 cell proliferation and particle degradation by lysosomal activation after endocytosis on J774 cell cultures were studied using confocal microscopy.

## 2. Results and Discussion

### 2.1. Characterization of TMC Nanoparticles and TMC/BSA Loaded Nanoparticles

**Figure 1:**
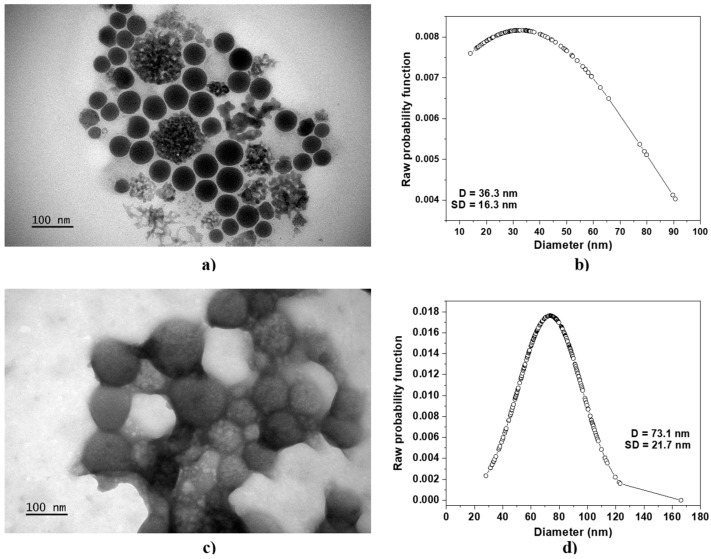
TEM micrographs of (**a**) TMC−NP and (**c**) TMC/BSA12−NP. The average diameter D and standard deviation SD of (**b**) TMC−NP and (**d**) TMC/BSA12−NP are shown. The line + dot corresponds to the diameters of the particles, measured on the micrograph.

Figure 1 shows the TEM micrographs of N-trimethyl chitosan nanoparticles without protein cargo, TMC−NP (Figure 1a), and TMC nanoparticles loaded with 12 μg/mL of BSA (TMC/BSA12−NP, Figure 1c), including their average diameter D and standard deviation SD (Figure 1b–d, respectively). 

Table 1 reports the average diameter D, standard deviation SD, and mean absolute deviation MAD of all fabricated samples. In all cases, the microfluidic fabricated nanoparticles showed a spherical shape with a coefficient variation ranging from 12.5 to 92.7%. The lower MAD corresponded to the TMC−NP, whereas the highest was TMC nanoparticles loaded with 55 μg/mL of BSA (TMC/BSA55−NP, Figure S1b). TMC nanoparticles loaded with 12 g/mL of BSA (TMC/BSA12–NP) or dye with FITC (TMC/BSA12_FITC−NP, Figure S1a) showed the same average diameter of about 70 nm. 

Huang et al. studied FITC-labeled and unlabeled chitosan nanoparticles with various molecular weights and degrees of deacetylation (DD) using the ionotropic gelation method with TPP. They observed that the mean diameter of 88% DD and Mw of 213 kDa FITC-labeled and unlabeled chitosan nanoparticles increased from 188 to 214 nm with a polydispersity of 0.43 and 0.36, respectively [12]. Xu et al. also contributed to the field, reporting the fabrication of TMC nanoparticles of 240 ± 42 nm [13]. In contrast, Amidi et al. fabricated TMC nanoparticles and ovalbumin (0.5 mg/mL)-loaded TMC nanoparticles of 360 ± 26 nm and 479 ± 32 nm (after purification), respectively, from chitosan of 42 kDa [8]. As previously reported by Majedi et al. and Tenorio et al., microfluidic techniques enable the fabrication of nanoparticles and their use as carriers [6,14]. 

Our research revealed that the concentration of BSA protein significantly affected the average diameter of the nanoparticles, with 12 mg/mL BSA-protein-producing nanoparticles with a MAD of 17.6% (TMC/BSA12−NP) and 55 mg/mL of BSA (TMC/BSA55−NP), leading to an increase to 92.7%. This suggests that the large dispersion in TMC/BSA55−NP is likely due to the aggregation of the BSA protein of various sizes acting as nucleation centers, resulting in a wide range of particle sizes. 

**Figure 2:**
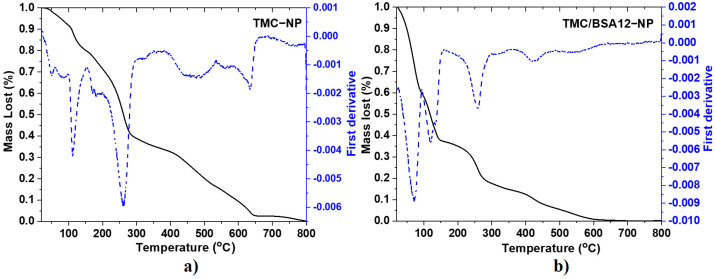
TGA (straight line) and first derivative (dash line) spectra of (**a**) TMC−NP and **(b**) TMC/BSA12−NP.

Figure 2 corresponds to the TGA and first derivative (DTGA) spectra of TMC−NP and TMC/BSA12−NP. Meanwhile, Table 2 and Figure S2 show the degradation stages and mass fraction of TMC/BSA55−NP nanoparticles, chitosan (CS), and TMC powders. The mass loss of chitosan (Figure S2a) starts at 30–120 °C, derived from the loss of ~4.7% moisture due to evaporation in and on the polymeric network. The second stage, at 185–355 °C, is attributed to the decomposition of chitosan due to the deacetylation and partial depolymerization of its chain [15]. In this second step of pyrolytic degradation of chitosan, with a mass loss of about 32%, the release of water from the polymer chain occurs, as well as of NH_3_, CO, CO_2_, and CH_3_COOH [16]. A third final stage of degradation occurs around the 630 °C peak, in the range of 450–750 °C, corresponding to the release of CH_4_ and the subsequent formation of a graphite-like structure upon calcination, with a mass loss of 58.7%. It depends on the degree of precursor deacetylation [15,16]. The TMC powder (Figure S2b) also shows water loss from 30 to 130 °C but with a lost mass of 20.4%. The temperature shift in this stage from 300 °C for CS powder to 165 °C in the case of TMC powder makes it less thermally stable. This can be attributed to incorporating the methyl terminals into the amine, which decreases intra-chain resistance [17].

The degradation of the various chains of the BSA protein is carried out in four main stages, with an approximate mass loss of 69%. The peaks of each stage are centered around 225, 313, 321, and 445 °C [18,19]. The fifth stage can be attributed to the water loss at approximately 65 °C. For the TMC−NP sample (Figure 2a), eight degradation ramps were observed, where the mass loss of the first three centered around 50, 85, and 112 °C was mainly due to the evaporation of water adsorbed and absorbed in the polymer network as well as volatile components of the TMC, present in the sample, being lower in mass than that corresponding to the lyophilized polymer. The second ramp of interest occurred at approximately 260 °C, but not at 165 °C as with TMC powder. The variation in thermal stability between the lyophilized TMC and the nanoparticulate sample (TMC−NP) can be attributed to the physical cross-linking that occurs between the positively charged sites of the N-trimethyl chitosan and the negatively charged sites of the sodium tripolyphosphate (TPP), during nanoparticle formation. This physical interaction thermally stabilizes the segments of the polymer chains of the nanoparticles [20,21]. The TGA of nanoparticles loaded with 12 μg of BSA, TMC/BA12−NP (Figure 2b) shows five degradation ramps. The peaks at 71, 120, and 133 °C indicate higher water retention within the polymer network of the TMC/BSA12−NP sample. In this case, the decomposition of TMC due to the partial depolymerization of the chitosan chain with the quaternation of the amino terminals also occurred around 259 °C, whereas the thermal decomposition ramps due to the presence of the BSA protein presented a shift in their DGTA peaks at 319, 422, and 553 °C. This is attributable to the electrostatic interaction between the terminals carboxyl and amine of TMC. The nanoparticles loaded with 55 μg of BSA in the TMC/BSA55−NP sample (Figure S2c) showed the same TMC/BSA12−NP sample degradation stages. Despite this, the BSA protein presented a higher shift in their DTGA peaks, probably due to the protein concentration.

**Figure 3:**
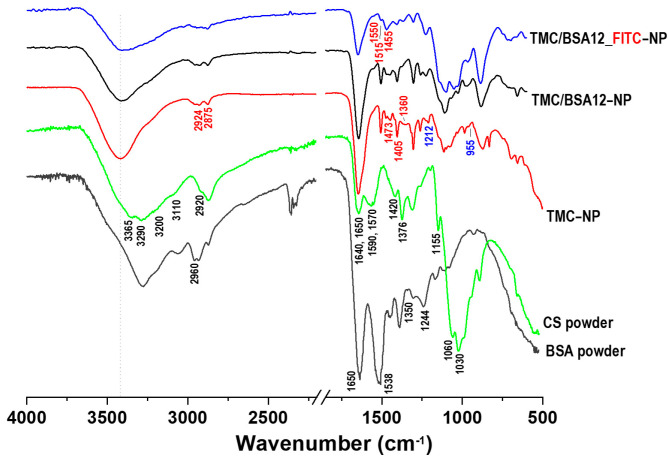
FTIR spectra of chitosan (CS) and bovine serum albumin (BSA) powders, TMC−NP, TMC/BSA12−NP, and TMC/BSA12_FITC−NP.

Figure 3 presents the FTIR spectra of CS and BSA powders, TMC−NP, TMC/BSA12−NP, and TMC/BSA12_FITC−NP samples. 

Chitosan powder (95%DD) presents the characteristics –OH stretching and intramolecular hydrogen bonds at 3365 and 3290 cm^−1^ and the asymmetric and symmetric –NH_2_ bonds at 3200 and 3110, respectively [22]. The C=O stretching bonds (acetamide groups) of amide I appear at 1650 cm^−1^, NH_2_ bending vibration of amine II at 1590 cm^−1^, C–N stretching, and N–H vibration of amide II are present at 1570 cm^−1^, whereas the bands of amide III at 1326 cm^−1^ are due to N–H and C–N stretching [12,22,23,24,25]. 

The TMC nanoparticle spectra showed a shifted shaper band at 3418 cm^−1^ associated with the combination of NH_2_ and OH bonds. As in CS, the band at 1650 corresponds to the C=O bond of amide I. However, its sharpness is due to the presence of the acetyl group and its methylated derivatives in the structure of chitosan. Amide II, which is mainly dependent on N–H bending, at 1590 cm^−1^ disappeared in TMC spectra owing to the replacement of hydrogen atoms by the alkyl group in the amino group. This band has been reported to decrease both TMC polymer and TMC nanoparticles [26]. The CH_3_ band appears at 1405 and 1360 cm^−1^ and the CH_2_ band at 1473 cm^−1^ owing to the substitution of methyl groups with H groups at the second carbon of chitosan (C_2_–NH_2_) [22,24]. The asymmetrical stretching of C–H bonds at 1455 cm^−1^ in the TMC–NP and TMC/BSA12−NP samples become strong because of the added N-alkyl groups during the methylation process, while in the TMC/BSA12_FITC−NP sample, it appears to be the strongest because of the added benzene ring stretching vibration of the FITC dye [26,27]. At 1212 y 955 cm^−1^, the P=O and P–O–P bonds were associated with the crosslinking between TPP and positively charged TMC sites during the nanoparticle fabrication [24]. In contrast, the BSA spectra show the C=O stretching band of amide I and the –NH bending of amide II bands of the α-helix at 1650 and 1538 cm^−1^, respectively, whereas the bands at 1350 and 1244–1240 cm^−1^ are attributed to the amide III of α-helix as well as to the antiparallel β-sheet vibrations [28,29,30]. The attenuation of the C=O stretching band (1650 cm^−1^) of TMC/BSA12−NP and TMC/BSA12_FITC−NP, as well as the intensity drop at 1538 cm^−1^, were related to the α-helix binding loss, and encapsulation of BSA [6,28]. Similarly, the disappearance of the 1244–1240 cm^−1^ band was attributed to modifications in the antiparallel β-sheet vibrations by TMC [29]. 

**Figure 4:**
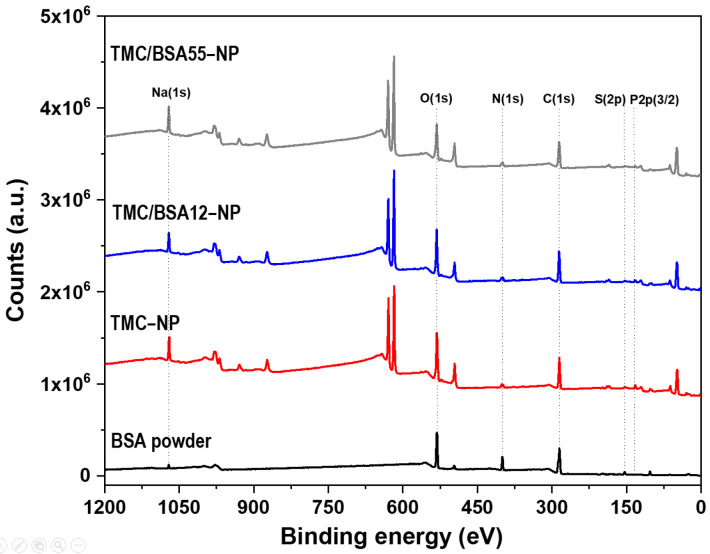
XPS surveys of bovine serum albumin (BSA), TMC−NP, TMC/BSA12−NP, and TMC/BSA12_FITC−NP.

The BSA protein (66 kDa) is a non-glycosylated globular protein of 583 amino acids and is organized in a single chain cross-linked with 17 cysteine residues in 8 disulfide bridges and a thiol group [31]. It is present in 60% of the plasma of vertebrate organisms and is structurally similar to human serum albumin (76%). XPS of TMC−NP, TMC/BSA12−NP, NP_TMC/BSA55−NP, and BSA protein were carried out in survey and detail modes to determine if the BSA protein was encapsulated during the microfluidic manufacturing process. Figure 4 shows the survey spectra of the binding energies of O(1s), N(1s), C(1s), P(2p3/2), and Na(1s) orbitals at 532, 399, 285, 132, and 1072 eV [32], respectively, present in the BSA, TMC, and TPP used in the synthesis of the nanostructures. Likewise, various binding energies above 600 eV were observed, associated with unwanted impurities derived from self-assembly on the glass substrates used during the characterization.

Table 3 summarizes the atomic percentages of each interest element in the samples and the C/O and N/C ratios. C(1s), O(1s), and N(1s) concentrations come from the structure of both BSA and TMC. Adventitious carbon also contributes to C(1s) quantification. The doublet of the S(2p) orbital around 163 eV was used as a marker since BSA protein and TMC contain nitrogen in their structures. The N/C and C/O ratios differ from those previously reported by Grenha et al. and Calvo et al. because of the synthesis process, the deacetylation degree of chitin (86% used by Grenha et al.), and the chitosan methylation. In this work, a TMC/TPP ratio = 1.6 was used compared to the ratios of 3.6 and 4.4 reported by the authors mentioned above [33,34].

**Figure 5:**
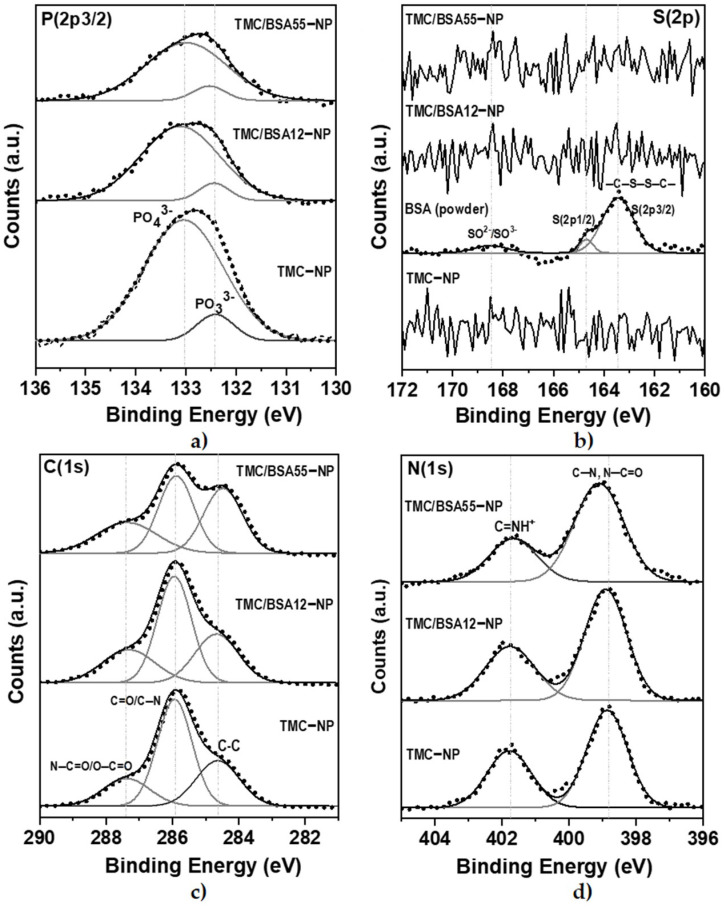
XPS detail spectra of TMC−NP, TMC/BSA12−NP, and TMC/BSA12_FITC−NP. (**a**) S(2p3/2); (**b**) C(1s); (**c)** N(1s); (**d**) O(1s) peaks. IgG protein was used as a control.

The deconvolution of the detailed spectra of the orbitals of P(2p3/2), S(2p), C(1s), and N(1s) are shown in Figure 5. The oxidation states PO_3_^3−^ y PO_4_^3−^ of P(2p3/2) orbital (Figure 5a) were present at 132.4 and 133.0 eV, respectively [35].

In the detailed spectrum of S(2p), Figure 5b, three signals are observed at 168.3, 164.7, and 163.4 eV, exclusively in the lyophilized BSA protein sample. The first corresponds to the oxidation states of sulfur SO^2−^ and SO^3−^, while at 164.7 and 163.4 eV, there is a doublet with the S(2p1/2) and S(2p3/2) states of the cystine disulfide bridges, C–S–S–C [35]. No sulfur signals were detected from TMC/BSA12−NP or TMC/BSA55−NP; therefore, the protein was encapsulated. Because XPS is a surface technique, the TMC thickness of the nanoparticles must be higher than 10 nm. Figure 5c shows the detailed spectra of the C(1s) orbital with the C–C/C–H bonds at 284.6 eV, C=O/C–N at 285.9 eV, and N–C=O/O–C=O at 287.3 eV [36,37]. Finally, Figure 5d shows the deconvolution of the N(1s) orbital, with the presence of C–N bonds at 398.8 eV and N–C=O/C=NH^+^ at 401.7 eV [35,37].

### 2.2. Effects of TMC-NP on Cell Proliferation

**Figure 6:**
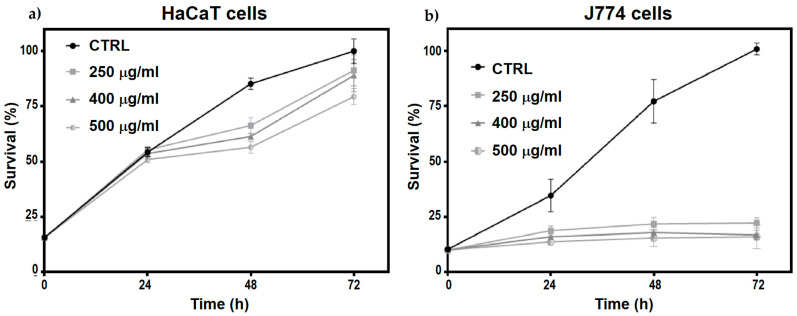
Temporal evolution (24, 48, and 72 h) of (**a**) HaCaT cells and (**b**) J774 cell proliferation treated with 250, 400, and 500 μg/mL of TMC/BSA12−NP.

The effects of N-Trimethyl chitosan nanoparticle treatment on cell proliferation were evaluated in HaCaT and J774 cell cultures. A significant decrease in J774 cell proliferation was observed after 24 h of treatment (Figure 6b), persisting up to 72 h without a trend toward recovery (multiple t-test, p < 0.05). However, in HaCaT cells, a slight decrease in cell proliferation was observed at 48 and 72 h post-treatment with the three different nanoparticle doses evaluated (multiple t-test, p < 0.05), with a trend towards proliferation recovery at 72 h (Figure 6a). 

These changes could be due to the inherent nature of the cells, as HaCaT cells belong to the keratinocyte lineage, whereas J774 cells belong to the monocyte lineage. Therefore, marked metabolic differences could link any cell cycle arrest with a change in metabolic activity or differentiation processes beyond possible cytotoxic effects.

The significant decrease in the proliferation rate of HaCaT cells treated with nanoparticles could be due to cytotoxic effects or the induction of differentiation processes. HaCaT cells show decreased proliferation during active differentiation, associated with the generation of inflammatory mediators involved in tissue remodeling and wound healing. This response may be linked to the activation of the innate immune system. To determine the particular cellular response stimulated by nanoparticles in these cells, it is necessary to perform specific assays that allow monitoring of the presence and levels of caspases related to apoptosis as well as some markers associated with the processes of inflammation and remodeling of the cellular matrix, such as CXCL8/IL8, VEGF, and MMP-9. Furthermore, assays to measure reactive oxygen species (ROS) and possible DNA damage would allow us to determine the cytotoxic properties of these nanoparticles [38,39].

When evaluating the toxicity of nanomaterials, the type of cells used for the assays, such as keratinocytes and macrophages, is crucial. Cell lines derived from immune response cells, particularly phagocytic cells, such as J774 and RAW264, exhibit specific responses to nanoparticle treatments, affecting their proliferation, size, and ROS content. Owing to their semi-adherent nature, J774 cells are more sensitive to nanoparticle treatment, which may decrease their anchorage and affect cell division. Generally, these effects, together with the participation of the growth factors GM-CSF and M-CSF, cause their differentiation to the functional states MO (non-activated), M1 (pro-inflammatory), or M2 (anti-inflammatory), each of which plays a distinct role in inflammation. To determine the differentiation state of J774 cells induced in response to nanoparticles, the presence of the CD80 and CD206 receptors, M1 and M2 differentiation markers, respectively, must be evaluated [40,41].

Table 4 shows the data of p values resulting from the multiple t-tests between the control, nanoparticle doses applied, and J774 and HaCaT cell cultures as a function of time. Considering the high phagocytic potential of J774 cells and the marked decrease in their proliferation caused by treatment with NPs (Table 4), these cells were selected to monitor the internalization of TMC/BSA12_FITC−NP by confocal microscopy and elucidate the metabolic process of its degradation.

### 2.3. Confocal Microscopy of Live Cells

**Figure 7:**
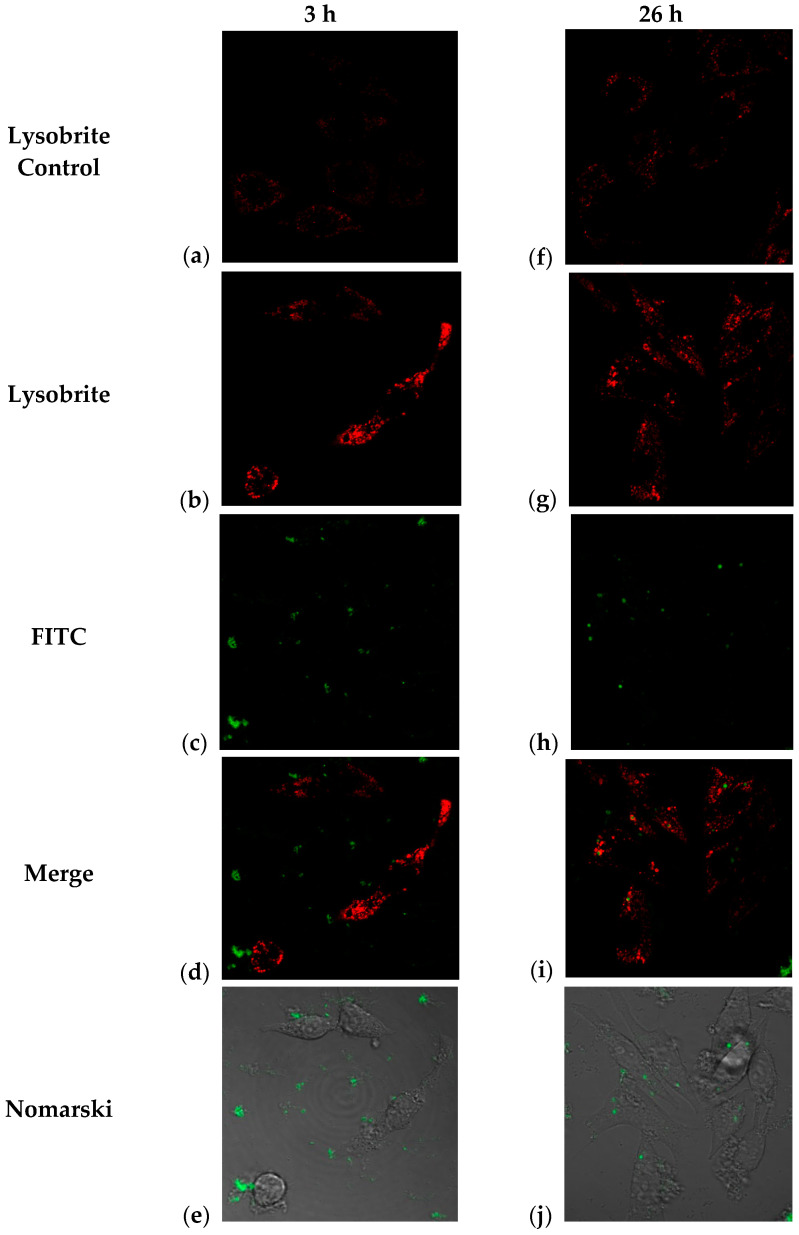
Confocal microscopy images corresponding to observations of J774 cells treated with TMC/BSA12_FITC-NP at 3 h (**a**–**e**) and 26 h (**f**–**j**); (**a**,**f**) control cells with no treatment and Lysobrite; (**b**,**g**) TMC/BSA12_FITC-NP treated cells and Lysobrite; (**c**,**h**) FITC channel of TMC-BSA12_FITC-NP treated cells; (**d**,**i**) Merge of images; (**e**,**j**) Nomarski observation of FITC channel and the structure of the J774 cells.

It is possible to determine the degradation routes of endocytosed particles and their cellular distribution through lysosomal activation assays. It has been reported [11] that J774 cells treated with fluorescent PLGA nanoparticles show an inflammatory response and colocalization with lysosomes. In this study, we performed two central tests: monitoring the intracellular trafficking of NPs containing BSA-FITC using Nomarski microscopy, where cellular morphology was observed, and lysosome staining with Lysobrite, whose mechanism of action is based on the pH-dependent activation of fluorescence in lysosomes (Figure 7). Therefore, the colocalization and increased intensity of the lysosomal marker indicate that treatment with these nanoparticles stimulates sustained lysosomal activity, suggesting that some pathways for their degradation occur in these compartments [42]. 

It is widely known that lysosomes contain numerous hydrolytic enzymes that degrade biological polymers, such as proteins, lipids, nucleic acids, and polysaccharides. These functions are tightly regulated by pH. Particle internalization can influence the composition of phagosomes and, consequently, the structure and maturation of phagolysosomes. The maturation process is accompanied by a progressive decrease in phagosomal pH, which drops from around pH 5.5 in nascent phagosomes to around pH 4.5 in lysosomes. This process occurs in most cell types, including “non–professional antigen-presenting cells” [43].

Lysosomal activation is closely related to phagocytic activity, indicating cargo transport from phagosomes to lysosomes for degradation. Because phagosomes form at the plasma membrane, they generally obtain membrane components from receptors involved in endocytosis, such as Fc receptors for immunoglobulin-bound particles and/or complement receptors for complement-bound particles. The maturation of phagosomes involves the sequential fusion of endosomes and lysosomes, progressively lowering the pH and ensuring the activity of hydrolytic enzymes. In macrophages, this process eliminates internalized microorganisms, while in dendritic cells, it prevents the complete degradation of antigen presentation [44].

In our study, as a first approach, we monitored the change in the location of the green label (BSA-FITC) and the red label (Lysobrite) in J774 cells treated with fluorescent NPs. After 3 h of treatment, the nanoparticles were located at the periphery of the cell (plasma membrane) (Figure 7b). Internalization began approximately 5 h after treatment, and this activity was maintained until the last observation at 26 h when we observed the highest number of particles inside the cells (Figure 7c,d). In addition, lysosomal activity was evaluated using Lysobrite red fluorochrome, the intensity of which depends on the metabolic activity of the lysosomes. Therefore, a semiquantitative analysis was performed using ImageJ software (Version 1.54j), and the numerical values of the pixels where the fluorescence signal was found in the cells were analyzed. Figure 7a,b shows that at 3 h after treatment, a clear difference in the intensity of red fluorescence (Lysobrite) was observed between the untreated control and cells treated with nanoparticles.

**Figure 8:**
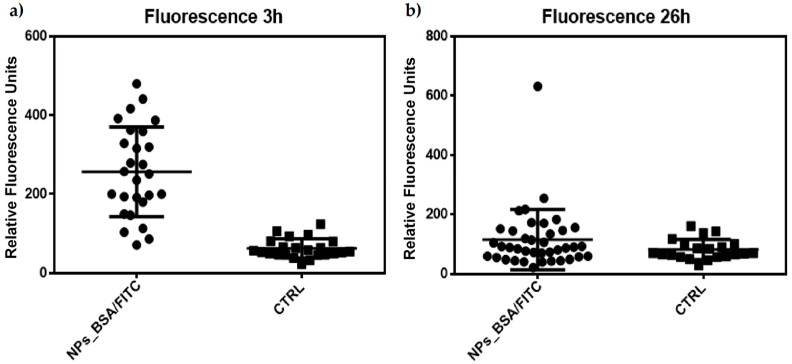
Semiquantitative analysis of the fluorescence data obtained from the confocal microscopy images from Figure 7. Data was analyzed using Mann–Whitney U for nonparametric data. (**a**) Significant difference between J774 cells treated with TMC/BSA/12_FITC nanoparticles at 3 h; (**b**) Non-significant difference between J774 cells treated with TMC/BSA_FITC nanoparticles at 26 h.

Semi-quantitative analysis of these cells (n = 23 control; n = 27 treated) indicates a statistically significant difference using a Mann–Whitney T-type comparison for non-parametric data (Figure 8a).

However, although this difference in fluorescence intensity appeared to be maintained 26 h after treatment (Figure 7f,g), the analysis (n = 21 controls; n = 39 treated) indicated that there is no difference between the treated and control cells (Figure 8b). These results may indicate a peak in lysosomal activation at the beginning of treatment with nanoparticles and that lysosomal activity stabilizes as time progresses, although its internalization prospers. Additionally, the acidification of phagosomes in the late stages of maturation is related to the activation of vacuolar ATPase (V-ATPase), as observed in the few focal points where the intensity of the red dye is higher. At the same time, an increase in pH stimulates lysozyme activation, as observed according to the decrease in the intensity of the red dye in the cells (Figure 7i). This could explain the behavior of our nanoparticles and the decrease in the fluorescence intensity in most of the lysosomes observed in Figure 7g [43].

The combination of the red and green channels corresponding to the detection of both fluorochromes by confocal microscopy indicated the co-localization of some marks at 26 h post-exposure. The above results show that not only is there an internalization of the nanoparticles that begins at 3 h post-exposure, but there is also a degradation of the material within the lysosomes around 26 h (Figure 7d–i). Interestingly, we also observed in Figure 7i,e, the green fluorescent mark goes from being dispersed to concentrated in clusters, as shown in Figure 7d,i; they go from being in the surroundings of the cell membrane and/or cytoplasm to colocalizing with lysosomal sacs. Colocalization was measured using the Pearson correlation and the Mander’s coefficient for the region of interest (ROI), with values ranging from 0.006 to 0.215 in the z-slices, with the central slices (7–9) presenting the highest values; the Mander’s coefficient was used to assess the pixel-intensity independent colocalization, and the central slice presented had values of M1 = 0.4, which is the fraction of the red pixels that overlap with green pixels, while M2 = 0.759 is the fraction of green pixels that overlap with red ones (Table S1) [42].

In this way, our observations in cells treated with trimethyl chitosan nanoparticles indicate that three hours after exposure, there is lysosomal activation, which probably indicates the beginning of endocytosis and activation of the phagosome, potentially increasing the expression of Fc or complement receptors. In comparison, at 26 h, the presence of “mature” phagosomes suggests active degradation of the nanoparticles and the fluorescent protein [11,43,44,45]. The endocytic pathway activated by nanoparticles in phagocytic and antigen-presenting cells is primarily determined by the surface properties of the NPs, which are influenced by their physicochemical properties, modifications, or protein aggregates on their surface, leading to more significant interaction with cells. 

It is essential to mention that depending on the behavior of lysosomes concerning the use of complement or Fc receptors, more precise data on the antigen presentation process could be obtained. In future formulations (such as vaccines), the expected behavior of a nanocarrier or an adjuvant is to stimulate the complement system minimally, have a controlled inflammatory response, and increase the production of MHC II molecules.

## 3. Materials and Methods

### 3.1. Materials

Chitosan medium molecular weight (CS, 190–310 kDa, DD85%, Sigma Aldrich, Burlington, MA, USA), Sodium Tripolyphosphate (TPP, Sigma-Aldrich), Bovine Serum Albumin (BSA, Sigma Aldrich), Dulbecco’s Modified Eagle’s Medium—High Glucose, and Dulbecco’s Modified Eagle’s Medium—F12, Fluorescein isothiocyanate (FITC) labeled BSA using the FITC labeling kit from Sigma Aldrich, Lysobrite Red (Cayman Chemicals, Ann Arbor, MI, USA), and Alamar Blue (ThermoFisher Scientific, Waltham, MA, USA) were used. The rest of the chemicals were reagent grade. J774 cells corresponded to the mouse macrophage cell line J774A.1 and were obtained from ATCC (TIB-67). HaCaT cells were generously granted by Dr. Norbert Fusenig [46].

### 3.2. TMC Synthesis

CS was deacetylated at 95% following the procedure described by (Martinou et al., 1995) [47]. Then, TMC was synthesized by methylation of chitosan using CH_3_I in the presence of NaOH, as described by (Sieval et al., 1998) [9]. Briefly, the system temperature was adjusted to 60 °C. Then, were mechanically mixed 80 mL of 1-methyl-2-pyrrolidone, 2 g of medium-molecular-weight chitosan (95% deacetylated), 4.8 g of sodium iodide, 11 mL of NaOH 15%, and 11.5 mL of methyl iodide for 1 h. The product was centrifuged at 3500 rpm for 10 min and washed with ethanol and diethyl ether. This process was repeated to maximize the yield. Subsequently, 2 mL of methyl iodide and 0.6 g of NaOH were added and mechanically mixed for 1 h. Then, 40 mL of 10% NaCl was added. The obtained polymer was centrifuged and washed as previously. The final product was freeze-dried for its use during nanoparticle synthesis.

### 3.3. TMC Nanoparticle Synthesis Using Microfluidic Technique

The TMC nanoparticle sample was synthesized using a microfluidic setup based on the methodology described by Tenorio et al. using water as a carried liquid [6]. An aqueous solution of 10 mg/mL of TMC and 6 mg/mL of TPP was prepared, placed in separate 50 mL conical tubes connected to the microfluidic setup, and pumped at a pressure of 1 bar through a T-shaped chip. Protein-loaded nanoparticles were synthesized similarly. In those cases, 12 mg/mL or 55 mg/mL of BSA, or 12 mg/mL of BSA labeled with FITC to the TMC solution, was added. In all cases, 1 mL aliquots were collected, freeze-dried, and weighed on an analytical balance to assess the mass–volume relation. Liquid and freeze-dried aliquots were used for subsequent characterization. Samples were labeled as TMC−NP, TMC/BSA12−NP, TMC/BSA55−NP, and TMC/BSA12_FITC−NP.

### 3.4. TMC Nanoparticle Characterization

The morphology, composition, thermal, and surface properties of the four types of TMC nanoparticles were characterized by transmission electron microscopy (TEM), Fourier transform infrared spectroscopy (FT-IR), thermogravimetric analysis (TGA), and X-ray electron spectroscopy (XPS). For TEM (JEOL-1400, Peabody, MA) observation, the samples were mounted on carbon-coated copper grids (400 mesh), stained with 2% uranyl acetate, and air-dried for at least 24 h. Their average diameter was determined through Image J^©^ software (Version 1.54j) over 150 sample particles. FTIR spectra were recorded in Alpha II equipment (Bruker, Billerica, MA, USA) from 525 to 4000 cm^−1^. Thermal properties (TGA 4000, Perkin Elmer, Waltham, MA, USA) were determined using 15 mg of the nanoparticle samples ranging from 20 °C to 800 °C at 15 °C/min. To XPS determine the elemental concentration and chemical ambient of selected samples, a 10 µL sample of each TMC−NP of interest was mounted on clean glass substrates and air-dried inside a desiccator for 24 h. Subsequently, survey (0–1200 eV) and detail spectra of S(2p), C(1s), N(1s), O(1s), and P(2p 3/2) were recorded (K-alpha, ThermoFisher Scientific equipment, Waltham, MA, USA).

### 3.5. Cell Culture, Proliferation Assay, and Endocytosis of TMC-NP

HaCaT cells were cultured using DMEM-F12 medium supplemented with 10% FBS incubated at 37 °C and 5% CO_2_. J774 cells were cultured using high-glucose DMEM supplemented with 10% FBS incubated at 37 °C and 5% CO_2_. Both types of cells were counted using a Neubauer chamber. For the proliferation assay with Alamar Blue, 12,000 HaCaT and 15,000 J774 cells were seeded in 48-well plates and allowed to adhere for 24 h. Cells were treated with 250, 400, and 500 µg/mL of BSA-loaded nanoparticles for 24, 48, and 72 h. At the end of each treatment, the medium was removed, the cells were washed with PBS, and then 500 μL of OptiMEM and 10% Alamar Blue were added and incubated for 1 h. The supernatant was collected in a 96-well plate and read using the Fluoroskan Ascent fluorometer with 570 and 600 nm filters. The endocytosis studies were carried out using 30,000 J774 cells seeded in p35 glass-bottom plates and allowed to adhere for 24 h. The cells were treated with 500 μg/mL of BSA-FITC-conjugated nanoparticles in a serum-free medium for 1 h. After this treatment, cells were washed with PBS, a fresh medium was added along with 50 μL of Lysobrite-Red (1:500 dilution), and allowed to stain for 30 min. Subsequently, the medium was removed, cells were washed with PBS, fresh medium containing 10% FBS was added, and they were observed using a confocal microscope (Nikon Eclipse Ti, Tokyo, Japan) during incubation at 37 °C and 5% CO_2_.

### 3.6. Statistical Analyses

The average diameters of nanoparticles and the proliferation and fluorescence comparison results are expressed as mean ± SD. The mean absolute deviation (MAD) of nanoparticles is given by the equation $\;MAD=\frac{\sum_{i=1}^{i=n}\left|x_{i}-\bar{x}\right|}{n}.$ The proliferation results were analyzed using a multiple t-test, and significance was determined using the Holm–Sidak method with α=5.000%; meanwhile, the fluorescence comparisons were analyzed using a Mann–Whitney U-Test for non-parametric data. In both cases, we used GraphPad Prism 6.00 for Windows (GraphPad Software, La Jolla, CA, USA, www.graphpad.com, accessed on 14 March 2024). The Pearson Correlation and the Mander’s Coefficient were calculated using the JACop plugin for FIJI by Image J [48,49]. 

## 4. Conclusions

TMC with and without BSA were successfully fabricated, with their size and dispersion depending on the concentration of the cargo protein. Their morphology, average diameter, dispersion, composition, and thermal stability indicated their potential use as nanocarriers since the cargo protein was encapsulated. The culture assays showed a significant difference between the proliferation of HaCaT and J774 cells treated with TMC/BSA12 nanoparticles, which could be related to the metabolic differences between each cell line. It was shown that J774 cells internalized the nanoparticles. In addition, a significant difference in lysosomal activation, 3 h post-treatment, between treated and control cells was found. Internalized nanoparticles colocalized with lysosomes from 8 h to 26 h.

## Figures and Tables

**Table 1:** Average diameter D, standard deviation SD, and mean absolute deviation MAD of fabricated nanoparticles.

Sample	Average Diameter, D [nm]	Standard Deviation, SD [nm]	Mean Absolute Deviation, MAD [nm]
TMC−NP	36.3	16.3	12.5
TMC/BSA12−NP	73.1	21.7	17.6
TMC/BSA12_FITC−NP	71.7	26.1	18.8
TMC/BSA55−NP	179.7	119.3	92.7

**Table 2:** Degradation stages and mass fraction of fabricated samples and CS and TMC powders.

Degradation Stages	Samples											
	CS (Powder)		TMC (Powder)		TMC−NP		TMC/BSA12−NP		TMC/BSA12_ FITC−NP		TMC/BSA55−NP	
	T [°C]	Mass Fraction	T [°C]	Mass Fraction	T [°C]	Mass Fraction	T [°C]	Mass Fraction	T [°C]	Mass Fraction	T [°C]	Mass Fraction
1st	62.9	4.7	104.6	20.4	49.3	0.5	70.8	16.8	61.2	13.7	52.1	0.8
2nd	303.4	31.6	165.9	45.4	84.5	2.6	119.9	28.5	114.0	33.7	113.7	5.1
3rd	630.0	58.7	281.0	69.0	111.6	4.9	133.0	32.0	190.1	50.2	181.9	10.4
4th	--	--	524.5	76.5	178.5	10.9	258.6	10.9	246.8	63.0	260.0	22.3
5th	--	--	800.0	85.0	262.1	22.9	319.2	22.9	323.7	71.8	437.4	33.4
6th	--	--	--	--	489.1	35.7	422.0	48.2	471.4	85.2	603.2	42.3
7th	--	--	--	--	552.6	39.1	800.0	53.3	600.0	100.0	800.0	44.0
8th	--	--	--	--	635.7	43.6	--	--	--	--	--	--

**Table 3:** Percentage concentration of the principal elements of fabricated samples.

Element	Concentration [%]		
	TMC−NP	TMC/BSA12−NP	TMC/BSA55−NP
S(2p)	0	0	0
C(1s)	33.2	39.1	40
N(1s)	5.4	6	6.2
O(1s)	57.9	52.7	52
P(2p3/2)	3.5	2.2	1.8
N/C ratio	0.162	0.153	0.155
C/O ratio	0.573	0.742	0.8

**Table 4:** Results of p values from the multiple t-tests between control and nanoparticle treatments on HaCaT and J774 cells.

Time (h)	CTRL vs. 250 µg (J774)	CTRL vs. 400 µg (J774)	CTRL vs. 500 µg (J774)	CTRL vs. 250 µg (HaCaT)	CTRL vs. 400 µg (HaCaT)	CTRL vs. 500 µg (HaCaT)
0	0.595543	0.525543	0.522477	0.839232	0.787912	0.97329
24	<0.0001	<0.0001	<0.0001	0.20449	0.690104	0.000638072
48	<0.0001	<0.0001	<0.0001	<0.0001	<0.0001	<0.0001
72	<0.0001	<0.0001	<0.0001	0.000380315	<0.0001	<0.0001

## Data Availability

The datasets presented in this article are not readily available because the data are part of an ongoing study. Requests to access the datasets should be directed to correspondence authors.

## Supplementary Materials

The following supporting information can be downloaded at: https://www.mdpi.com/article/10.3390/molecules29153621/s1, Table S1. Statistical values collected from the Person correlation and the Mander’s coefficient used to analyze the colocalization of nanoparticles with lysosomes. Figure S1. Average diameter D and standard deviation SD of (a) TMC/BSA12_FITC-NP and (b) TMC/BSA55-NP. Figure S2. TGA (straight line) and first derivative (dash line) spectra of (a) CS powder, (b) TMC powder, and (c) TMC/BSA5-NP.

## References

[1] Kumar B.P., Chandiran I.S., Bhavya B., Sindhuri M. (2011). Microparticulate drug delivery system: A review. Indian J. Pharm. Sci. Res..

[2] Rizvi S.A.A., Saleh A.M. (2018). Applications of nanoparticle systems in drug delivery technology. Saudi Pharm. J..

[3] Gupta R.K., Siber G.R. (1995). Adjuvants for human vaccines-current status, problems and future prospects. Vaccine.

[4] Reed S.G., Orr M.T., Fox C.B. (2013). Key roles of adjuvants in modern vaccines. Nat. Med..

[5] Kumacheva E. (2011). Microfluidic Reactors for Polymer Particles.

[6] Tenorio-Barajas A.Y., Olvera M.d.l.L., Romero-Paredes G., Altuzar V., Garrido-Guerrero E., Mendoza-Barrera C. (2023). Chitosan, Chitosan/IgG-Loaded, and N-Trimethyl Chitosan Chloride Nanoparticles as Potential Adjuvant and Carrier-Delivery Systems. Molecules.

[7] Peniche H., Acosta N. (2003). Chitosan: An Attractive Biocompatible Polymer for Microencapsulation. Macromol. Biosci..

[8] Amidi M., Romeijn S.G., Borchard G., Junginger H.E., Hennink W.E., Jiskoot W. (2006). Preparation and characterization of protein-loaded N-trimethyl chitosan nanoparticles as nasal delivery system. J. Control. Release.

[9] Sieval A.B., Thanou M., Kotzé A.F., Verhoef J.C., Brussee J., Junginger H.E. (1998). Preparation and NMR characterization of highly substituted N-trimethyl chitosan chloride. Carbohydr. Polym..

[10] Jearanaiwitayakul T., Sunintaboon P., Chawengkittikul R., Limthongkul J., Midoeng P., Chaisuwirat P., Warit S., Ubol S. (2021). Whole inactivated dengue virus-loaded trimethyl chitosan nanoparticle-based vaccine: Immunogenic properties in ex vivo and in vivo models. Hum. Vaccines Immunother..

[11] Nicolete R., Santos D.F.D., Faccioli L.H. (2011). The uptake of PLGA micro or nanoparticles by macrophages provokes distinct in vitro inflammatory response. Int. Immunopharmacol..

[12] Huang M., Khor E., Lim L.Y. (2004). Uptake and Cytotoxicity of Chitosan Molecules and Nanoparticles: Effects of Molecular Weight and Degree of Deacetylation. Pharm. Res..

[13] Xu J., Liu C., Xu Y., Shan W., Liu M., Huang Y. (2015). Mechanism of cellular uptake and transport mediated by integrin receptor targeting trimethyl chitosan nanoparticles. Yao Xue Xue Bao Acta Pharm. Sin..

[14] Majedi F.S., Hasani-Sadrabadi M.M., Hojjati Emami S., Shokrgozar M.A., Vandersarl J.J., Dashtimoghadam E., Bertsch A., Renaud P. (2013). Microfluidic assisted self-assembly of chitosan based nanoparticles as drug delivery agents. Lab Chip.

[15] Szymańska E., Winnicka K. (2015). Stability of chitosan—A challenge for pharmaceutical and biomedical applications. Mar. Drugs.

[16] Corazzari I., Nisticò R., Turci F., Faga M.G., Franzoso F., Tabasso S., Magnacca G. (2015). Advanced physico-chemical characterization of chitosan by means of TGA coupled on-line with FTIR and GCMS: Thermal degradation and water adsorption capacity. Polym. Degrad. Stab..

[17] Pardeshi C.V., Belgamwar V.S. (2016). Controlled synthesis of N,N,N-trimethyl chitosan for modulated bioadhesion and nasal membrane permeability. Int. J. Biol. Macromol..

[18] Adamiano A., Lesci I.G., Fabbri D., Roveri N. (2015). Adsorption of bovine serum albumin onto synthetic Fe-doped geomimetic chrysotile. J. R. Soc. Interface.

[19] Jones A., Zeller M.A., Sharma S. (2013). Thermal, mechanical, and moisture absorption properties of egg white protein bioplastics with natural rubber and glycerol. Prog. Biomater..

[20] Facchi S.P., Scariot D.B., Bueno P.V.A., Souza P.R., Figueiredo L.C., Follmann H.D.M., Nunes C.S., Monteiro J.P., Bonafé E.G., Nakamura C.V. (2016). Preparation and cytotoxicity of N-modified chitosan nanoparticles applied in curcumin delivery. Int. J. Biol. Macromol..

[21] Sadeghi A., PourEskandar S., Askari E., Akbari M. (2023). Polymeric Nanoparticles and Nanogels: How Do They Interact with Proteins?. Gels.

[22] Ding Y., Xia X.H., Zhang C. (2006). Synthesis of metallic nanoparticles protected with N,N,N-trimethyl chitosan chloride via a relatively weak affinity. Nanotechnology.

[23] Zając A., Hanuza J., Wandas M., Dymińska L. (2015). Determination of N-acetylation degree in chitosan using Raman spectroscopy. Spectrochim. Acta Part A Mol. Biomol. Spectrosc..

[24] Geçer A., Yıldız N., Çalımlı A., Turan B. (2010). Trimethyl chitosan nanoparticles enhances dissolution of the poorly water soluble drug Candesartan-Cilexetil. Macromol. Res..

[25] Liu Y., Shen X., Zhou H., Wang Y., Deng L. (2016). Chemical modification of chitosan film via surface grafting of citric acid molecular to promote the biomineralization. Appl. Surf. Sci..

[26] Brito L.A., Malyala P., O’Hagan D.T. (2013). Vaccine adjuvant formulations: A pharmaceutical perspective. Semin. Immunol..

[27] Jiang L., Li X., Liu L., Zhang Q. (2013). Cellular uptake mechanism and intracellular fate of hydrophobically modified pullulan nanoparticles. Int. J. Nanomed..

[28] Ahmad F., Zhou Y., Ling Z., Xiang Q., Zhou X. (2016). Systematic elucidation of interactive unfolding and corona formation of bovine serum albumin with cobalt ferrite nanoparticles. RSC Adv..

[29] Sripriyalakshmi S., Anjali C.H., Doss C.G.P., Rajith B., Ravindran A. (2014). BSA nanoparticle loaded atorvastatin calcium—A new facet for an old drug. PLoS ONE.

[30] Yadav P., Yadav A.B. (2021). Preparation and characterization of BSA as a model protein loaded chitosan nanoparticles for the development of protein-/peptide-based drug delivery system. Future J. Pharm. Sci..

[31] Topală T., Bodoki A., Oprean L., Oprean R. (2014). Bovine serum albumin interactions with metal complexes. Clujul Med..

[32] Gorham J. (2012). NIST X-ray Photoelectron Spectroscopy Database—SRD 20 (1.0.4) [Dataset].

[33] Calvo P., Remuñán-López C., Vila-Jato J.L., Alonso M.J. (1997). Novel hydrophilic chitosan-polyethylene oxide nanoparticles as protein carriers. J. Appl. Polym. Sci..

[34] Grenha A., Seijo B., Serra C., Remuñán-López C. (2007). Chitosan nanoparticle-loaded mannitol microspheres: Structure and surface characterization. Biomacromolecules.

[35] Siow K.S., Britcher L., Kumar S., Griesser H.J. (2018). XPS study of sulfur and phosphorus compounds with different oxidation states. Sains Malays..

[36] Foster R.N., Harrison E.T., Castner D.G. (2016). ToF-SIMS and XPS Characterization of Protein Films Adsorbed onto Bare and Sodium Styrenesulfonate-Grafted Gold Substrates. Langmuir.

[37] Munir A., Haq T.U., Qurashi A., Rehman H.U., Ul-Hamid A., Hussain I. (2019). Ultrasmall Ni/NiO nanoclusters on thiol-functionalized and-exfoliated graphene oxide nanosheets for durable oxygen evolution reaction. ACS Appl. Energy Mater..

[38] Colombo I., Sangiovanni E., Maggio R., Mattozzi C., Zava S., Corbett Y., Fumagalli M., Carlino C., Corsetto P.A., Scaccabarozzi D. (2017). HaCaT Cells as a Reliable In Vitro Differentiation Model to Dissect the Inflammatory/Repair Response of Human Keratinocytes. Mediat. Inflamm..

[39] Nabeshi H., Yoshikawa T., Matsuyama K., Nakazato Y., Tochigi S., Kondoh S., Hirai T., Akase T., Nagano K., Abe Y. (2011). Amorphous nanosilica induce endocytosis-dependent ROS generation and DNA damage in human keratinocytes. Part. Fibre Toxicol..

[40] Feito M.J., Casarrubios L., Oñaderra M., Gómez-Duro M., Arribas P., Polo-Montalvo A., Vallet-Regí M., Arcos D., Portolés M.T. (2021). Response of RAW 264.7 and J774A.1 macrophages to particles and nanoparticles of a mesoporous bioactive glass: A comparative study. Colloids Surf. B Biointerfaces.

[41] Orekhov A.N., Orekhova V.A., Nikiforov N.G., Myasoedova V.A., Andrey V. (2019). Monocyte differentiation and macrophage polarization. Vessel. Plus.

[42] Elsabahy M., Wooley K.L. (2012). Design of polymeric nanoparticles for biomedical delivery applications. Chem. Soc. Rev..

[43] Dunn K.W., Kamocka M.M., McDonald J.H. (2011). A practical guide to evaluating colocalization in biological microscopy. Am. J. Physiol. -Cell Physiol..

[44] Blander J.M., Medzhitov R. (2006). On regulation of phagosome maturation and antigen presentation. Nat. Immunol..

[45] Kunzmann A., Andersson B., Thurnherr T., Krug H., Scheynius A., Fadeel B. (2011). Biochimica et Biophysica Acta Toxicology of engineered nanomaterials: Focus on biocompatibility, biodistribution and biodegradation. BBA Gen. Subj..

[46] Boukamp P., Petrussevska R.T., Breitkreutz D., Hornung J., Markham A., Fusenig N.E. (1988). Normal keratinization in a spontaneously immortalized aneuploid human keratinocyte cell line. J. Cell Biol..

[47] Martinou A., Kafetzopoulos D., Bouriotis V. (1995). Chitin deacetylation by enzymatic means: Monitoring of deacetylation processes. Carbohydr. Res..

[48] Schindelin J., Arganda-Carreras I., Frise E., Kaynig V., Longair M., Pietzsch T., Preibisch S., Rueden C., Saalfeld S., Schmid B. (2012). Fiji: An open-source platform for biological-image analysis. Nat. Methods.

[49] Bolte S., Cordelières F.P. (2006). A guided tour into subcellular colocalization analysis in light microscopy. J. Microsc..

