# Supplementary material for: Lysosomal Activation Mediated by Endocytosis in J774 Cell Culture Treated with N-Trimethyl Chitosan Nanoparticles
Source: Molecules. 2024 Jul 31;29(15):3621. doi: 10.3390/molecules29153621 (PMC11313802; doi:10.3390/molecules29153621)
Supplement: Supplementary file 1 [file molecules-29-03621-s001.zip › Table S1.pdf]

Table S1. Statistical values collected from the Person correlation and the Mander's coefficient used to analyze the colocalization of nanoparticles with lysosomes.

Slice	Pearson correlation value	Mander's coefficient M1	Mander's coefficient M2
**Z1**	0.052	0.072	0.494
**Z2**	0.073	0.142	0.626
**Z3**	0.102	0.219	0.724
**Z4**	0.109	0.269	0.758
**Z5**	0.148	0.312	0.788
**Z6**	0.169	0.353	0.788
**Z7**	0.197	0.383	0.788
**Z8**	0.198	0.4	0.759
**Z9**	0.215	0.418	0.711
**Z10**	0.212	0.409	0.649
**Z11**	0.180	0.394	0.565
**Z12**	0.136	0.381	0.478
**Z13**	0.074	0.344	0.389
**Z14**	0.032	0.325	0.331
**Z15**	0.006	0.29	0.274
