# Supplementary figures and images for: Lysosomal Activation Mediated by Endocytosis in J774 Cell Culture Treated with N-Trimethyl Chitosan Nanoparticles
Source: Molecules. 2024 Jul 31;29(15):3621. doi: 10.3390/molecules29153621 (PMC11313802; doi:10.3390/molecules29153621)

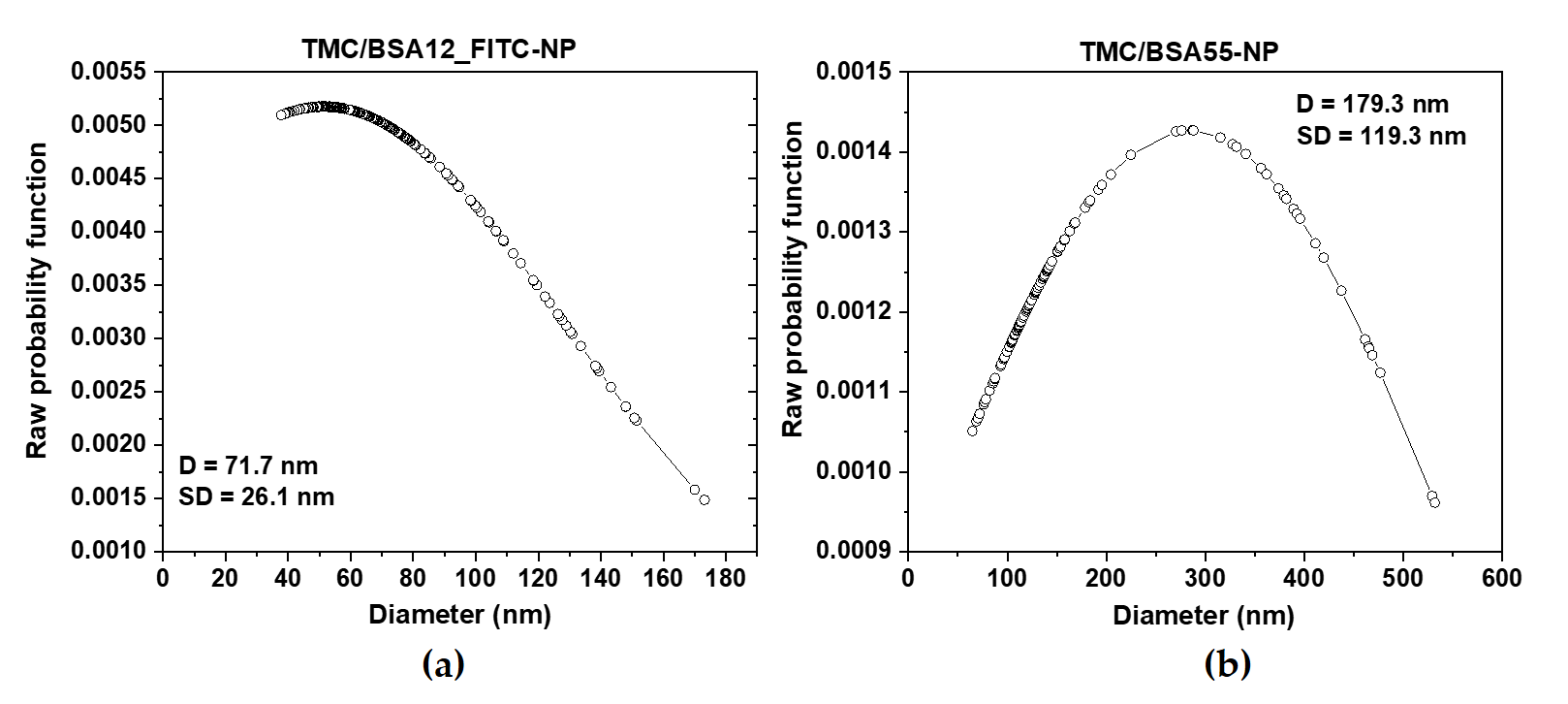

Supplement: Supplementary file 1 [file molecules-29-03621-s001.zip › Figure S1.png]

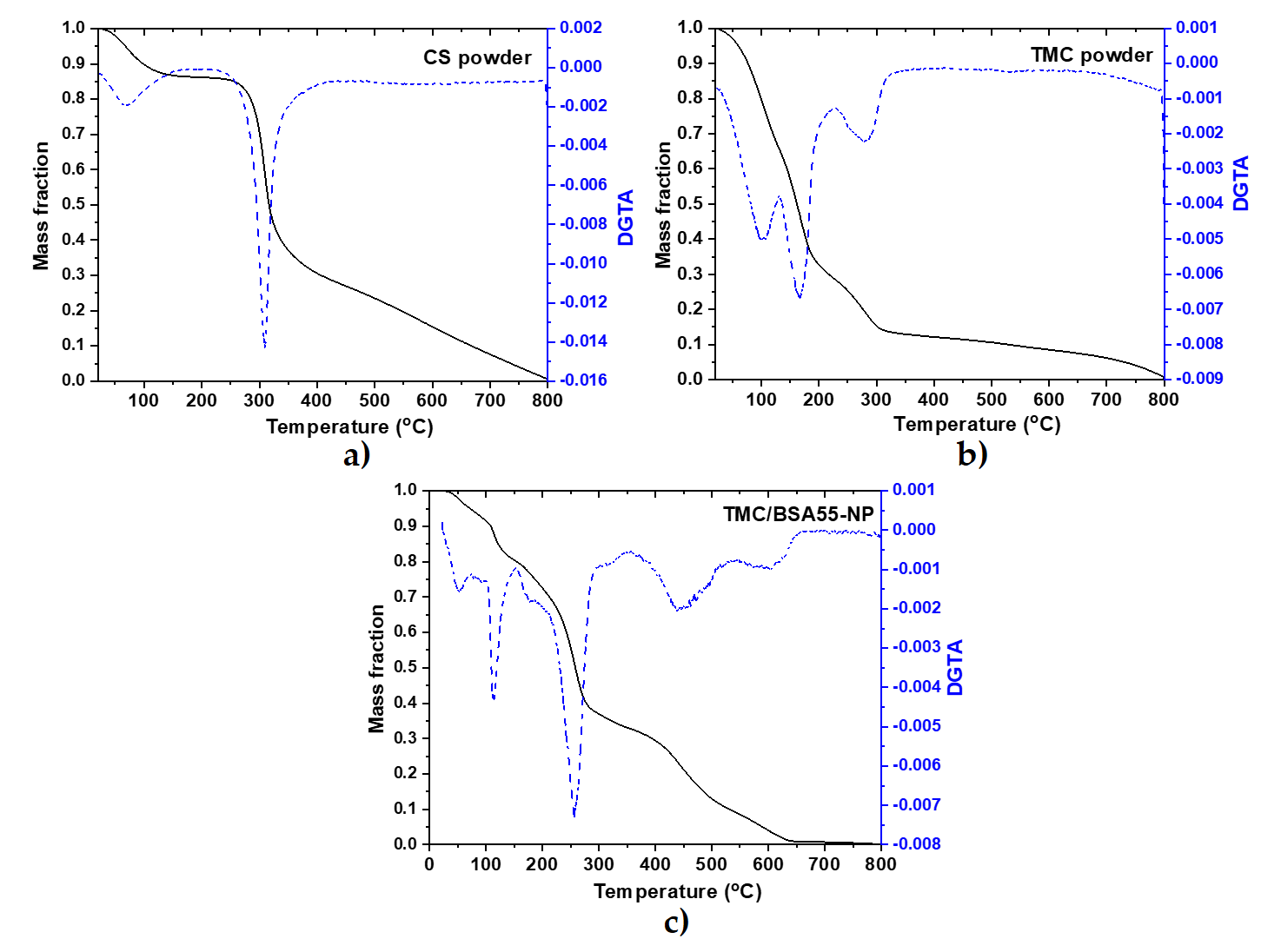

Supplement: Supplementary file 1 [file molecules-29-03621-s001.zip › Figure S2.tif]
